# Dielectric Characteristics and Microwave Absorption of Graphene Composite Materials

**DOI:** 10.3390/ma9100825

**Published:** 2016-10-13

**Authors:** Kevin Rubrice, Xavier Castel, Mohamed Himdi, Patrick Parneix

**Affiliations:** 1 DCNS Research Technocampus Océan, 5 rue de l’Halbrane, 44340 Bouguenais, France; patrick.parneix@dcnsgroup.com; 2 Institut d’Electronique et de Télécommunications de Rennes (IETR, UMR-6164)/Institut Universitaire de Technologie de Saint-Brieuc/Université de Rennes 1, 18 rue Henri Wallon, 22004 Saint-Brieuc & 263 avenue du Général Leclerc, 35042 Rennes, France; xavier.castel@univ-rennes1.fr (X.C.); mohamed.himdi@univ-rennes1.fr (M.H.)

**Keywords:** multifunctional composite materials, graphene, physical properties, analytical modeling, electromagnetic absorption, complex dielectric constants

## Abstract

Nowadays, many types of materials are elaborated for microwave absorption applications. Carbon-based nanoparticles belong to these types of materials. Among these, graphene presents some distinctive features for electromagnetic radiation absorption and thus microwave isolation applications. In this paper, the dielectric characteristics and microwave absorption properties of epoxy resin loaded with graphene particles are presented from 2 GHz to 18 GHz. The influence of various parameters such as particle size (3 µm, 6–8 µm, and 15 µm) and weight ratio (from 5% to 25%) are presented, studied, and discussed. The sample loaded with the smallest graphene size (3 µm) and the highest weight ratio (25%) exhibits high loss tangent (tanδ = 0.36) and a middle dielectric constant ε′ = 12–14 in the 8–10 GHz frequency range. As expected, this sample also provides the highest absorption level: from 5 dB/cm at 4 GHz to 16 dB/cm at 18 GHz.

## 1. Introduction

Graphene is made out of a two-dimensional carbon structure in hexagonal lattice with a nanosizing in the perpendicular direction. It presents interesting thermal, mechanical, and electrical properties [1] along with light weight and high electrical characteristics: electron mobility µ = 230,000 cm^2^·V^−1^·s^−1^ [2] and conductivity σ (σ = 400 S·m^−1^ in the powder form [3] and σ = 5 × 10^6^ S·m^−1^ in thin layer form [4]). Another graphene characteristic has been highlighted in recent years: its microwave absorption and electromagnetic shielding abilities. Different materials with graphene particles have therefore been studied [5,6,7,8] and show interesting electromagnetic absorption feature. For example, the use of chemically reduced graphene oxide with residual defects improves the electromagnetic wave absorption through additional relaxation processes, namely dielectric, dipole, and polarization relaxations. Microwave reflection loss as much as −6.9 dB is then obtained at 7 GHz [5]. Graphene-polymethylmethacrylate nanocomposite microcellular foams present high electromagnetic interference shielding efficiency (13–19 dB at 8–12 GHz) by favoring multireflections and scattering of the incident microwaves into the foam samples [6]. Graphene sheets with polyaniline nanorods embedded in a paraffin matrix show microwave reflection loss below −20 dB from 7.0 GHz to 17.6 GHz by improving the Debye relaxation process [7]. Graphene nanoplatelets in epoxy resin exhibit −14.5 dB maximum reflection loss at 18.9 GHz, mainly attributed to the charge multipoles at the polarized interfaces into the composite material [8].

The present paper deals with graphene particles embedded into an epoxy resin for microwave absorption applications, and more specifically for the microwave isolation of closer antennas attached on the same composite panel and working between 2 GHz and 18 GHz. It is organized as follows. In Section 2, sample fabrication and the measuring process are described. In the following section, the composite materials loaded with graphene are investigated in light of two parameters: the graphene particle size and the weight ratio of graphene particles into the composite materials. For each material and parameter combination, the complex permittivity was measured from 2 GHz to 18 GHz, and the results are discussed. The microwave absorption ability of the composite materials from 4 GHz to 18 GHz is also investigated and related to the dielectric constant and loss tangent results. Finally, conclusions are drawn in Section 4.

## 2. Materials and Methods

### 2.1. Graphene Composite Material Fabrication

Samples were fabricated from pre-dispersals of graphene particles in epoxy resin and a related hardener (masterbatch). The graphene particles consist of the agglomeration of single-layer graphene flakes, each about 0.34-nm-thick corresponding to the interlayer spacing of graphite from sp2 carbon chemistry [9]. Graphene is obtained through mechanical exfoliation of small pieces of graphite. Three different masterbatches were used, preloaded with 3-µm, 6–8-µm, and 15-µm graphene particle sizes with respective weight ratios of 25%, 15%, and 10% (masterbatches manufactured and supplied by the Nanovia company, Louargat, France, with Prime™ 27 epoxy resin and Prime™ 20 hardener from the Gurit company, Newport, UK). The final weight ratio of graphene in each sample is adjusted after the mixture of masterbatches (preloaded resin + preloaded hardener) with pristine epoxy resin and pristine hardener (dilution with 28:100 hardener/resin weight ratio). This process is particularly well adapted (I) to obtain fair particle dispersion; (II) to prevent any graphene aggregation; and finally (III) to produce a homogeneous material [10]. Epoxy resin has been selected for this study due to its low viscosity (480–510 cP for Prime™ 27 epoxy resin and 13–15 cP for Prime™ 20 hardener @ 25 °C), allowing a high graphene ratio, its low exothermicity during the cross-linking reaction, and its low shrinkage characteristic. Numerous samples (a total of 24) with different particle sizes and weight ratios have been fabricated (Table 1). A standard 5-mm-thick sample dimension was selected. It promotes both measurements to assess the complex permittivity and the microwave absorption. The reference sample is a pristine epoxy resin free of graphene particles.

### 2.2. Measuring Process

**Figure 1:**
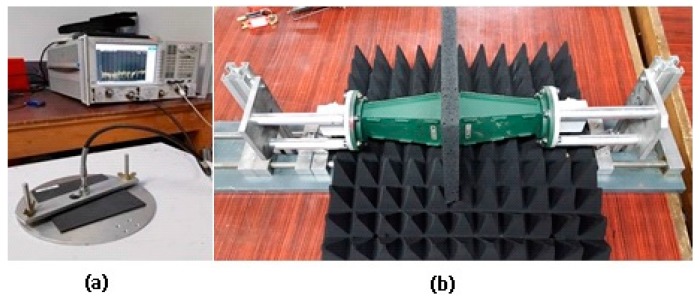
Dielectric kit measurement (**a**) & free-space bench (**b**).

The samples were characterized over a large broadband frequency by assessing three parameters: the complex permittivity, the material reflection, and the material absorption factors. The real and imaginary parts of the complex permittivity ε* (ε* = ε′ + jε″) were retrieved from the S_11_ reflection coefficient measured via a commercial dielectric kit (High Temperature probe (19 mm in diameter) associated with the N1500A Material Measurement Suite software, both supplied by the Keysight Technologies company, Les Ulis, France). The set-up consisted in a probe in close contact with the surface sample. After an air/short/water probe calibration, results between 2 GHz and 18 GHz were presented to be consistent with the forthcoming reflection and absorption characterizations. To restrict any implementation error and microwave cable effect, a homemade bench-test was fabricated (Figure 1a). At least 4 measuring points were checked on each sample. The average of the values provided the dielectric characteristics of the sample. The standard deviation of the 4 measuring points was ε′ = 1.0 and tanδ = 0.05. The accuracy was then equal to ±5% for the dielectric constant ε′ and ±0.05 for the loss tangent (tanδ = ε″/ε′). A smooth sample surface without any porosity is essential to the reliability of the measurement, due to the close contact requirement between the kit probe and the sample under test.

The second and third studied parameters here are the microwave reflection and absorption of the materials. To this end, 3 pairs of high matching horn antennas (4.65–8 GHz // 8–12 GHz // 12.5–18 GHz) were successively used to measure the S-parameters in a near-field setting. The largest apertures of the horn antennas (109.25 mm × 79 mm) had imposed the sample dimensions (150 mm × 150 mm, see Table 1). This choice was to be explained by the lack of lens to focus the electromagnetic waves on the samples at frequencies as low as 4 GHz. Prior to sample measurements, a preliminary transmission calibration between horn antennas in pairs was carried out. Then, each sample was positioned in close contact between the two horn apertures to restrict diffraction phenomena from the sample edges (Figure 1b). On the one hand, we considered the samples as infinite planes with a thickness d. On the other hand, S-parameters were affected by the near-field transmission setting, which induced phase shift effects and then oscillations on such microwave measurements. Time domain and gating techniques were applied to reduce unwanted reflections. It is worth noting too that the use of a single broadband antenna (2–18 GHz) must be avoided here because a high-sensibility reflection was required to measure the material absorption with accuracy. Recording the reflection S_11_ and the transmission S_21_ coefficients, the material absorption was computed from the linear power conservation Equation (1) expressed as follows (Equation (2) [11]):

(1) $${\left|S_{11}\right|}^{2}+{\left|S_{21}\right|}^{2}+\left|absorption\right|=1;$$

(2) $$\alpha_{dB}=10\log\left(1-{\left|S_{11}\right|}^{2}\right)-10\log\left({\left|S_{21}\right|}^{2}\right).$$

Using the sample thickness (Table 1), the material absorption was normalized in dB/cm, namely α. This method is valuable for any kind of materials with reflectors, absorbers, or dielectric behaviors.

## 3. Results and Discussion

### 3.1. Complex Permittivity Results

#### 3.1.1. Influence of the Particle Size

**Figure 2:**
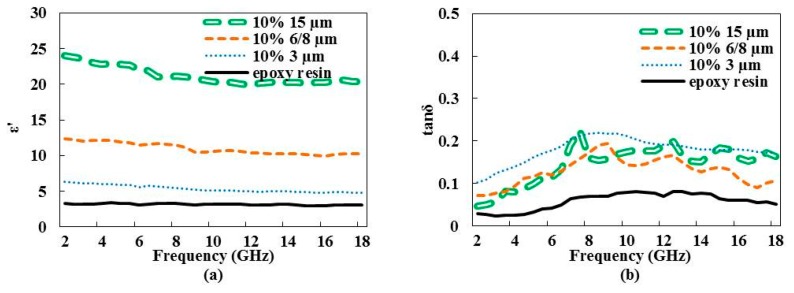
Variation of dielectric constant ε′ (**a**) and loss tangent tanδ (**b**) vs. frequency of epoxy resin loaded with different graphene particle sizes at a constant weight ratio (10%). Pristine epoxy resin values are given for reference.

Samples loaded with graphene particles of different sizes at a fixed weight ratio (10%) were first studied. Figure 2a presents the variation of the dielectric constant up to 18 GHz. At a constant working frequency, the more particle size increases, the more dielectric constant increases. Increasing working frequency leads to a decrease of the dielectric constant due to relaxation phenomena, such as rotational and vibrational transitions, electronic, and atomic polarizations, as mentioned by Griffiths [12] and Atwater et al. [13].

Figure 2b shows the nonlinear variation of the loss tangent parameter versus frequency. Loss tangent increases up to 8–10 GHz, behavior also noted from composite materials loaded with graphene or carbon nanotubes without any satisfactory explanation [14,15,16]. Sample loaded with the 3-µm particle size exhibits the highest loss tangent value.

#### 3.1.2. Influence of the Weight Ratio

**Figure 3:**
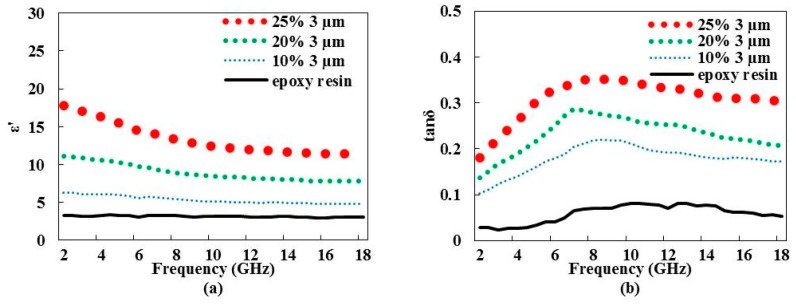

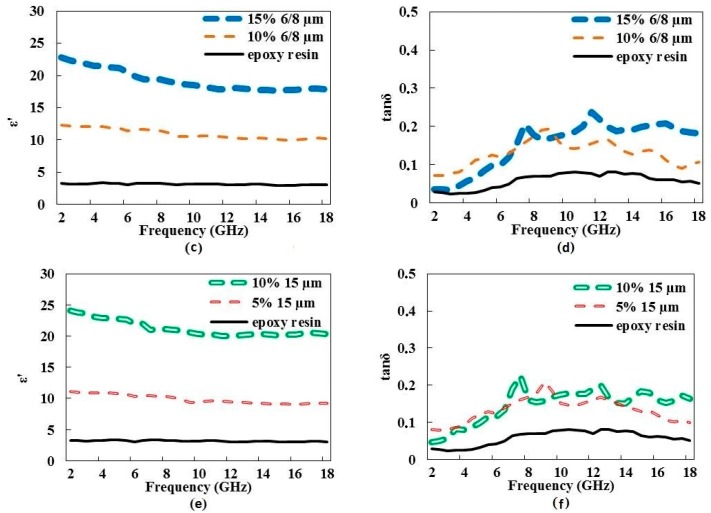
Variation of dielectric constant ε′ and loss tangent tanδ vs. frequency of epoxy resin loaded with a 3-µm graphene particle size (**a**,**b**); a 6–8-µm graphene particle size (**c**,**d**); and a 15-µm graphene particle size (**e**,**f**) with various weight ratios, respectively. Pristine epoxy resin values are given for reference.

Other samples fabricated with different weight ratios and the three particle sizes (3 µm, 6–8 µm, and 15 µm) were studied (Figure 3). Maximum weight ratio achieved depends on the graphene size. For example, epoxy resin loaded with the 3-µm graphene particle size cannot exceed 25% weight ratio, while that loaded with the 15-µm particle size achieves a maximum of 10%. For higher weight ratio values, the mixture becomes extremely viscous and lumped in consistency. Accordingly, the supplier cannot insure the mixture quality. Figure 3a,b present the complex permittivity variation versus the frequency of samples loaded with the 3-µm graphene particle size. Results show the increase of the dielectric constant (Figure 3a) and of the loss tangent (Figure 3b) values with respect to the weight ratio. Furthermore, the maximum loss tangent value of this study was reached and set at 0.36 in the 8–10 GHz frequency band. The weight ratio variation versus frequency is similar to that of the particle size variation (see Section 3.1.1): with increasing frequency at a constant weight ratio, the dielectric constant decreases, while the loss tangent increases up to 8–10 GHz and decreases afterwards. Dielectric constant values of composite materials loaded with the largest particle sizes (Figure 3c,e) remain higher than ~9. The related loss tangent does not improve much, even at higher weight ratios (Figure 3d,f).

#### 3.1.3. Particle Size and Weight Ratio Twofold Influence

**Figure 4:**
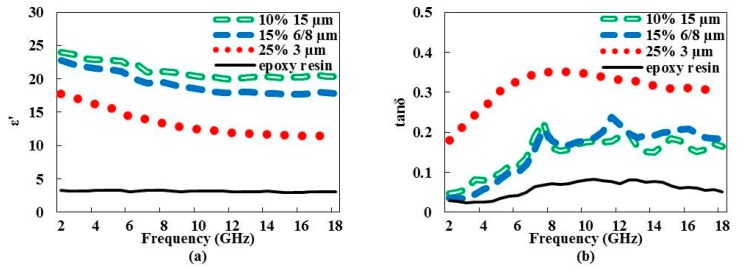
Variation of dielectric constant ε′ (**a**) and loss tangent tanδ (**b**) vs. frequency of epoxy resin loaded with the maximum weight ratio of the related graphene particle sizes. Pristine epoxy resin values are given for reference.

Samples loaded with the largest particle sizes at the maximum weight ratio exhibit closer dielectric constant and loss tangent values (Figure 4). The use of the smallest graphene particles (3 µm) promotes higher loss tangent values (Figure 4b). According to Chung [17], an electromagnetic absorption mechanism can result from the electric dipoles of materials with high dielectric constant values. The interaction between the electromagnetic field and the material induces molecular movements, charge relaxation, or both, leading to energy dissipation [18,19]. The use of the smallest graphene particles with the highest weight ratio increases the interaction surface between graphene and the matrix and enhances the absorption mechanism by the composite material and thus the loss. This behavior has also been highlighted by Crane et al. through their study on the metal particle size effect on microwave absorption [19].

### 3.2. Electromagnetic Absorption

The assessment of the electromagnetic absorption was made using an homemade near field free-space bench as described in Section 2.2. S-parameter measurements provide the electromagnetic reflected power |S_11_|^2^ and the electromagnetic transmitted power |S_21_|^2^ by and through the composite material under test, respectively. With Equation (2), it is easy to compute the electromagnetic absorption α of the composite material.

#### 3.2.1. Influence of the Particle Size

**Figure 5:**
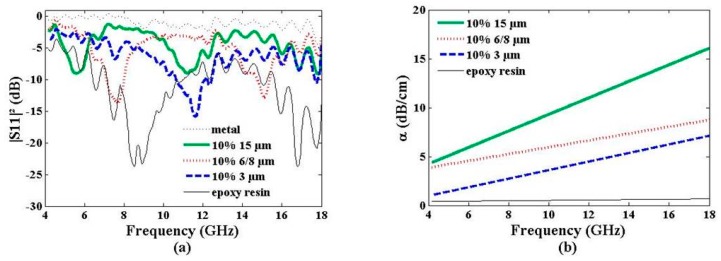
Reflected power |S_11_|^2^ (**a**) and electromagnetic absorption α (**b**) vs. frequency of epoxy resin loaded with different graphene particle sizes at a constant weight ratio (10%). Pristine epoxy resin values are given for reference.

Electromagnetic absorption of the graphene composite materials depends on different parameters. The influence of the graphene particle size in epoxy resin was first investigated. Figure 5 shows the reflected power of the 10% weight ratio graphene composites loaded with 3 µm, 6–8 µm, and 15 µm particle sizes. Two additional curves have been plotted for reference: the pristine dielectric epoxy resin and a perfect reflector (a metal plate). Compared with a perfect reflector (|S_11_|^2^ ≈ 0 dB), |S_11_|^2^ values of the composite materials remain lower than −3 dB in the 8–18 GHz frequency range, evidencing the penetration of the electromagnetic wave into the materials. Reflected power and complex permittivity are closely interrelated with respect to Equation (3) applied with a normal incident electromagnetic wave in air striking a dielectric slab, as follows [20]:

(3) $${\left|S_{11}\right|}_{dB}^{2}=10\;\log{\left(\left|e^{-2j\beta d}\times \frac{\sqrt{\varepsilon \prime \left(1-tan\delta \right)}-\sqrt{\varepsilon \prime_{air}\left(1-tan\delta_{air}\right)}}{\sqrt{\varepsilon \prime \left(1-tan\delta \right)}+\sqrt{\varepsilon \prime_{air}\left(1-tan\delta_{air}\right)}}\right|\right)}^{2},$$

where β = 2π/λ is the wavenumber, (ε′_air_ = 1, tanδ_air_ = 0) is the dielectric characteristics of air, and (ε′, tanδ) is the dielectric characteristics of the composite material with thickness d.

The variation of ε′ between 5 and 24 (Figure 2a) and tanδ between 0.05 and 0.22 (Figure 2b) induces a variation of |S_11_|^2^ between −0.8 dB and −6.4 dB, as observed in Figure 5a. Observation of resonance peaks close to 10 GHz with |S_11_|^2^ ≤ −10 dB is explained by the total reflection of the electromagnetic waves by the composite material, the guide wavelength λ_g_ into the composite material (λ_g_ = λ/$\sqrt{\varepsilon \prime }$) satisfying the following Equation (4) [20]:

(4) $$n\times \frac{\lambda_{g}}{2}=d,$$

where *n* is a positive integer (d = 5 mm for the composite materials and d = 10 mm for the pristine epoxy resin, see Table 1). The pristine epoxy resin sample shows a resonance at ~9 GHz (*n* = 1); the sample loaded with a 3-µm graphene particle size presents a resonance peak at 11.7 GHz (*n* = 1); those with 6-8-µm graphene particle size at 7.6 GHz (*n* = 1) and 15.0 GHz (*n* = 2); those with 15 µm at 5.7 GHz (*n* = 1) and 11.4 GHz (*n* = 2)—in agreement with the increase of the ε′ value when the graphene particle size increases (Figure 2a).

Figure 5b presents the electromagnetic absorption trend curves of the samples over the 4–18-GHz frequency band extracted from the near field free-space bench measurements. Absorption variation follows the same trend for all samples: weak values at low frequency (1 dB/cm for the 3-µm graphene particle size and 4 dB/cm for the 15-µm graphene particle size at 4 GHz) and stronger ones at high frequency (7 dB/cm for the 3-µm graphene particle size and 16 dB/cm for the 15-µm graphene particle size at 18 GHz). Note that the pristine epoxy resin exhibits a constant absorption value (~0.1 dB) over the full working frequency band.

#### 3.2.2. Influence of the Weight Ratio

**Figure 6:**
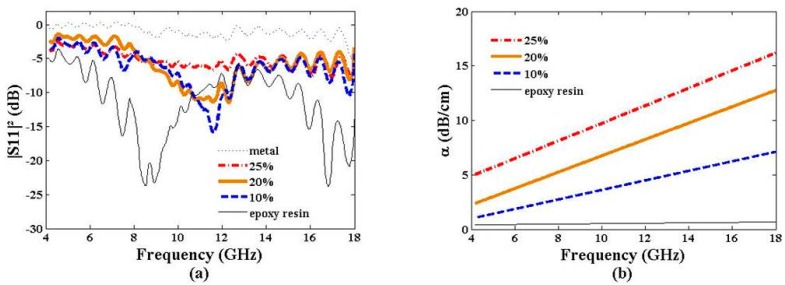
Reflected power |S_11_|^2^ (**a**) and electromagnetic absorption α (**b**) vs. frequency of epoxy resin loaded with a 3-µm graphene particle size with various weight ratios. Pristine epoxy resin values are given for reference.

The second investigated parameter is the graphene weight ratio in epoxy resin. Figure 6a presents the reflected power of the 3-µm graphene composite materials loaded with 10%, 20%, and 25% weight ratio. The perfect reflector and pristine epoxy resin curves have also been plotted for reference. The behavior of the reflected power is similar to that studied in Section 3.2.1, in agreement with the related complex permittivity values. The 10% weight ratio sample shows a resonance peak at 11.7 GHz (*n* = 1); those with 20% weight ratio at 10.8 GHz (*n* = 1). The magnitude peak is damped by the increase of the graphene weight ratio, due to the rising of the related tanδ value (Figure 3b). It is worth noting that the resonance peak is invisible for the 25% weight ratio sample, exhibiting the highest loss tangent of the present study (tanδ = 0.36).

Figure 6b presents the electromagnetic absorption trend curves of the samples over the 4–18-GHz frequency band. Absorption variation follows the same trend as that observed in Figure 5b: 1 dB/cm for 10% weight ratio sample and 5 dB/cm for 25% weight ratio sample at 4 GHz; 7 dB/cm for 10% weight ratio sample and 16 dB/cm for 25% weight ratio sample at 18 GHz.

**Figure 7:**
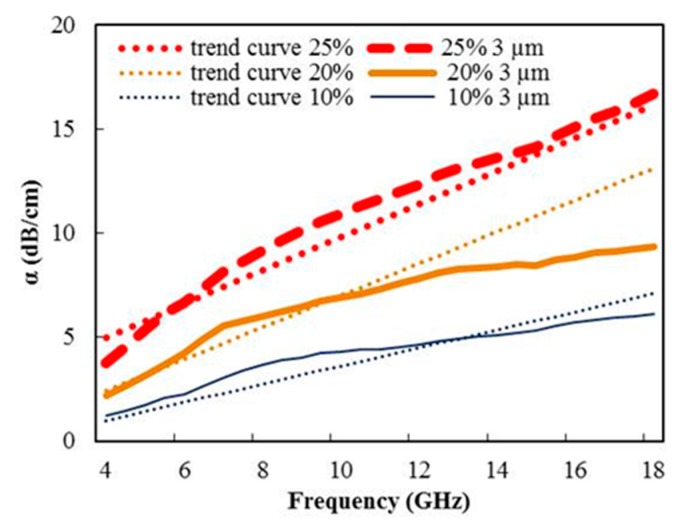
Electromagnetic absorption α vs. epoxy resin loaded with a 3-µm graphene particle size with various weight ratios.

As expected, high dielectric constant and loss tangent values promote high electromagnetic absorption α, as described by Equation (5) from [20]:

(5) $$\alpha =A\times \frac{\omega }{c}\sqrt{\frac{{µ}^{\prime }\varepsilon^{\prime }}{2}}\times \sqrt{\left[\sqrt{1+{\left(tan\delta \right)}^{2}}-1\right]},$$

where ω is the working pulsation; c is the speed of light in vacuum; µ′ is the relative permeability of the composite material (µ = 1); (ε′, tanδ) is the dielectric characteristics of the composite material; A is an adjustment factor (A = 8.68/100 dB·Np^−1^) to convert α in dB/cm. In order to check the relevance of the ε′ and tanδ values provided by the dielectric kit method (Figure 3a,b), α values have been computed from Equation (5) and compared with the measured values deduced from Equation (2). The satisfactory fit between the measured and computed α values confirms the reliability of the dielectric characteristics (ε′, tanδ) (Figure 7), despite a drift between the trend curve and the measured values for the 20% weight ratio sample at the frequency band edge. The frequency dependence of Equation (5) also explains the linear variation of α versus the working frequency.

#### 3.2.3. Particle Size and Weight Ratio Twofold Influence

**Figure 8:**
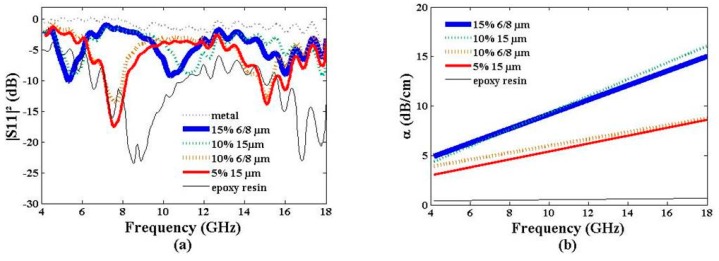
Reflected power |S_11_|^2^ (**a**) and electromagnetic absorption α (**b**) vs. frequency of epoxy resin loaded with 6–8-µm and 15-µm graphene particle sizes with various weight ratios. Pristine epoxy resin values are given for reference.

These parameters also have an influence on the reflected power (Figure 8a) and the electromagnetic absorption (Figure 8b). The first relevant point concerns the resonance peaks (|S_11_|^2^ ≤ −10 dB). Samples with a 6–8-µm graphene particle size and 10% weight ratio shows two resonance peaks at 7.6 GHz (*n* = 1) and 15.0 GHz (*n* = 2). With the increase in the weight ratio to 15%, these peaks shift at 5.6 GHz (*n* = 1) and 10.5 GHz (*n* = 2). This behavior is consistent with that of samples loaded with a 15-µm graphene particle size at 5% and 10% weight ratios. The second point is a behavior similar to these samples. The reflected power of a sample loaded with a 6–8-µm graphene particle size with a 10% weight ratio and that of a sample loaded with a 15-µm graphene particle size with a 5% weight ratio are alike. In the same way, a sample loaded with a 6-8-µm graphene particle size with a 15% weight ratio presents a similar |S_11_|^2^ to that loaded with a 15-µm graphene particle size with a 10% weight ratio. We attribute these results to the close dielectric constants and loss tangents of the two sets of samples (Table 2).

Between 4 GHz and 18 GHz, the absorption α ranges from 3–4 dB/cm at 4 GHz to 8.7 dB/cm at 18 GHz for the 6–8-µm graphene particle size with 10% weight ratio and the 15-µm graphene particle size with 5% weight ratio samples. After a relative weight ratio increase, α ranges from 4–5 dB/cm at 4 GHz to achieve 15–16 dB/cm at 18 GHz for the 6–8-µm graphene particle size with 15% weight ratio and the 15-µm graphene particle size with 10% weight ratio samples.

**Figure 9:**
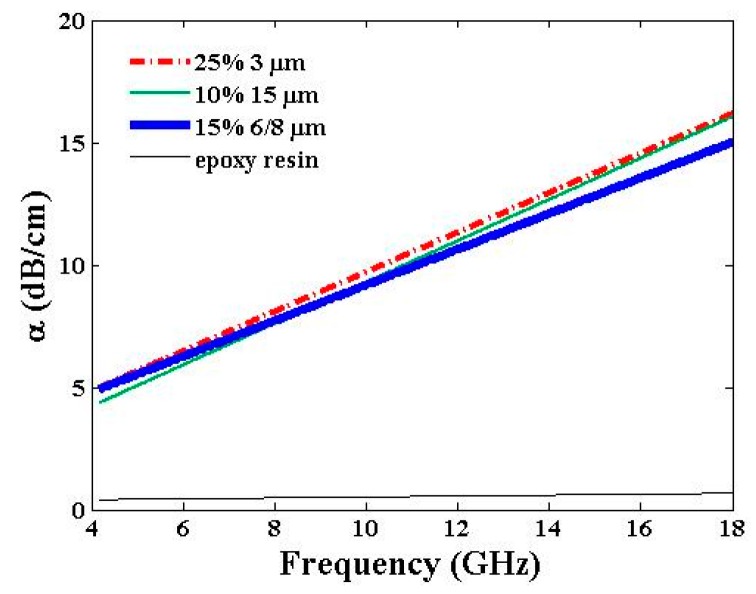
Electromagnetic absorption α vs. frequency of samples loaded with the maximum weight ratio of the related graphene particle sizes. Pristine epoxy resin values are given for reference.

Figure 9 presents the absorption responses of the samples loaded with the maximum weight ratio for the three graphene sizes. The three samples exhibit high dielectric constants (Figure 4a) with differing loss tangent values (Figure 4b). It is worth noting that the lower ε′ value of the 3-µm graphene particle size with a 25% weight ratio sample is compensated by a higher tanδ value, and therefore explains the sameness of the three absorption responses. Finally, at 10 GHz, a minimum absorption of 9 dB/cm is reached with all samples.

## 4. Conclusions

In this study, graphene particles were used as filler in a thermosetting resin, namely an epoxy resin, in view of microwave absorption and electromagnetic shielding applications. The composite materials produced have thereby been evaluated in relation to two relevant parameters: the graphene particle size (3 µm, 6–8 µm, and 15 µm) and the graphene weight ratio (ranging from 5% to 25%). For these, the dielectric constant and loss tangent (ε′, tanδ), and the microwave absorption α of each sample, were measured using a dielectric kit (from 2 GHz to 18 GHz) and a homemade near field free-space bench (from 4 GHz to 18 GHz), respectively. The epoxy resin loaded with the smallest graphene size (3 µm) and the highest weight ratio (25%) exhibits the highest loss tangent (tanδ = 0.36) and a middle dielectric constant value (ε′ ≃ 12–14) in the 8–10 GHz frequency range. Filling the epoxy resin with the smallest graphene particles promotes high loss tangent at microwaves and thus the microwave absorption of the electromagnetic waves by the composite material. Moreover, a lower dielectric constant will be favored to restrict the impedance mismatch at the interface between air and surface samples and thus to restrict the electromagnetic wave reflectivity at this interface. The composite material then provides a high absorption level: from 5 dB/cm at 4 GHz to 16 dB/cm at 18 GHz.

## Figures and Tables

**Table 1:** Sample details.

Sample Size (mm^3^) (Length × Width × Thickness)	Graphene Size (µm)	Weight Ratio (%)
150 × 150 × 10	0	0
150 × 150 × 5	3	10/20/25
150 × 150 × 5	6–8	10/15
150 × 150 × 5	15	5/15

**Table 2:** Dielectric constant and loss tangent of epoxy resin loaded with 6–8-µm and 15-µm graphene particle sizes with various weight ratios.

Particle Size (µm)	Weight Ratio (%)	ε′ @10 GHz	tanδ @10 GHz	ε′ @18 GHz	tanδ @18 GHz
6–8	10	10.6	0.14	10.2	0.11
15	5	9.5	0.15	9.2	0.10
6–8	15	18.5	0.18	17.8	0.18
15	10	20.4	0.18	20.4	0.16
